# Serum 25-Hydroxyvitamin D Is Inversely Associated with Nasopharyngeal Carcinoma: A Hospital-Based Matched Case–Control Study in Malaysia

**DOI:** 10.3390/nu16030397

**Published:** 2024-01-30

**Authors:** Vaidehi Ulaganathan, Munn Sann Lye, Su Peng Loh, Yoke Yeow Yap, Mirnalini Kandiah, Digsha Augundhooa, Tanima Bhattacharya, Ebtesam Al-Olayan, Chuanyi Wang

**Affiliations:** 1 Department of Food Science and Nutrition, Faculty of Applied Sciences, UCSI University, Kuala Lumpur 43000, Malaysia; kandiahmirnalini607@gmail.com (M.K.); digshaaugund@gmail.com (D.A.); 2 Department of Community Health, Faculty of Medicine and Health Sciences, Universiti Putra Malaysia, Serdang 43400, Selangor, Malaysia; 3 Department of Nutrition and Dietetics, Faculty of Medicine and Health Sciences, Universiti Putra Malaysia, Serdang 43400, Selangor, Malaysia; sploh@medic.upm.edu.my; 4 Graduate School of Medicine, KPJ Healthcare University, Nilai 71800, Negeri Sembilan, Malaysia; yokeyeow@gmail.com; 5 Otorhinolaryngology, KPJ Johor Specialist Hospital, Johor Bharu 80100, Johor, Malaysia; 6 Faculty of Applied Science, Lincoln University College, Petaling Jaya 47301, Selangor, Malaysia; btanima1987@gmail.com; 7 Department of Zoology, College of Science, King Saud University, Riyadh 11495, Saudi Arabia; eolayan@ksu.edu.sa; 8 School of Environment Science and Engineering, Shaanxi University of Science and Technology, Xi’an 710021, China; wangchuanyi@sust.edu.cn

**Keywords:** serum 25(OH)D, vitamin D, nasopharyngeal carcinoma, case–control, Malaysia

## Abstract

Serum 25(OH)D deficiency consistently demonstrated molecular mechanisms through which chronic inflammation is associated with the risk of nasopharyngeal carcinoma (NPC). This study aimed to determine the association between serum 25(OH)D and NPC. A matched case–control study was conducted at two local hospitals. A total of 300 histologically confirmed NPC cases were matched with controls for age, gender, and ethnicity, and assessed for vitamin D status and other nutritional factors. Mean Vitamin D concentration was significantly lower among cases compared to controls (63.17 ± 19.15 nmol/L and 67.34 ± 23.06 nmol/L) (*t* = −2.41, *p* = 0.016). Multiple conditional logistic regression analysis indicated that higher levels of serum 25(OH)D were associated with reduced odds of NPC (AOR = 0.73, 95% CI = 0.57–0.94, *p* = 0.016) controlling for confounders including BMI, physical activity, smoking status, alcohol consumption, consumption of food high in vitamin D, salted fish consumption, and family history of NPC. There was a significant association between inadequate serum 25(OH)D status with accumulation of four risk factors and increased odds of getting NPC using polynomial regression analysis. Increased NPC odds ratios were observed after sequential accumulation of additional risk factors with the presence of inadequate serum 25(OH)D status (OR = 0.54, 95% CI = 0.27, 4.77, *p* = 0.322, OR = 1.04, 95% CI = 0.64, 1.72, *p* = 0.267, OR = 1.15, 95% CI = 0.73, 1.80, *p* = 0.067, OR = 1.93, 95% CI = 1.13, 3.31, *p* = 0.022, and OR = 5.55, 95% CI = 1.67, 10.3, *p* < 0.001 respectively). Future research in Malaysia should involve both prospective cohort studies and randomized controlled trials to confirm and further clarify the role of vitamin D in NPC outcomes.

## 1. Introduction

Nasopharyngeal carcinoma (NPC) develops in the nasopharynx, commonly in the fossa of Rosenmuller [1]. Although GLOBOCAN has reported that the worldwide incidence of NPC is 1 per 100,000 people per year, it is higher in certain populations [2]. In Malaysia, out of 48,639 new cancer cases diagnosed in 2020, NPC was ranked as the fourth most common cancer, with 2222 (4.6%) new cases reported [3]. NPC is more common among Chinese males, with a cumulative incidence of 1.1 per 100,000 [4]. NPC affects men more than women, with a ratio of 3:1 [4]. The differences in geographic and ethnic incidences of NPC suggest that genetic factors may play a large role. Preventing cancer is a crucial and effective strategy to decrease both cancer incidence and mortality. Notably, the positive impact of immunonutrition on antitumor effects has been linked to enhanced quality of life and improved survival among NPC cases [5]. The role of vitamin D has gained attention in recent years for the prevention of diverse types of cancer: colorectal [6], breast [7], prostate [8], and head and neck cancers [9]. It is hypothesized that vitamin D prevents cancer because it is anti-inflammatory, anti-proliferative, and promotes apoptosis [10]. At the same time, evidence shows it enhances the function of the innate immune system, and it may induce immune tolerance [11]. Thus, exploring potential associations between vitamin D and NPC risk and survival holds considerable significance. 

Vitamin D is fat-soluble, which enables our body to store any extra amount [12]. Vitamin D can be obtained directly from the sun as well as from diet. There are few natural dietary sources of vitamin D such as liver and fatty fish; however, these days, most foods are fortified with vitamin D [13]. Among vitamin D-rich foods commonly consumed in Malaysia are beef liver, pork, fish, egg, soy products, and dairy products [14]. Beef liver is a good source of vitamin D, providing 49 IU, while pork contains 32 IU per 100 g. The amount of vitamin D in farm animals depends on the vitamin D concentration in the feed and also exposure to the sun [15]. Fish is an excellent source of vitamin D, with approximately 1000 IU per 100 g. Vitamin D is significantly higher in fatty fish such as mackerel and tuna [16]. An egg contains 52 IU of vitamin D, especially in the egg yolk. Nowadays, vitamin D-fortified eggs are available in the market, which contain between 200 IU/egg to 8000 IU/egg depending on the amount of vitamin D-enriched foods the hens receive [14]. Recently, the Food and Drug Administration has approved an increase in vitamin D fortification in dairy milk and milk alternatives, which adds up to 205 IU per serving of vitamin D available from these foods. Food fortification with vitamin D has resulted in a significant improvement in serum 25(OH)D concentration [13].

The prevalence of vitamin D deficiency has tripled since the 1980s and has stimulated clinical and public health research to quantify the optimal amount of vitamin D as a biomarker for better health outcomes [12]. This trend may be related to low sunlight exposure, the use of sunscreen, and changes in dietary habits [17]. Although 1,25-Dihydroxy vitamin D is the biologically active form of vitamin D, most researchers prefer to estimate deficiency using serum 25-hydroxy vitamin D (25(OH)D) as it is the best indicator of vitamin D status, has a longer circulating half-life (15 days), and has much higher concentration in blood. Serum 25(OH)D reflects vitamin D produced in the skin from sunlight and absorbed from diet [17].

Vitamin D moderates the effect of obesity, physical inactivity, and other lifestyle factors such as smoking, alcohol consumption, and environmental exposures that contribute to the risk of developing cancer, especially in rarer cancers such as endometrial [18], esophageal [19], gastric [20], kidney [21], ovarian [22], and pancreatic cancers [23], as well as non-Hodgkin lymphoma [24]. A multicentered case–control study in Hong Kong found that genetically low serum 25(OH)D tripled the risk of NPC [25]. A study in Wuhan reported an inverse association between serum 25(OH)D and NPC risk (OR = 0.97; 95% CI: 0.96–0.99; *p* = 0.004) [26], and no significant association between vitamin D receptor gene polymorphisms and NPC [27]. Although their studies suggest a plausible association, the evidence is mainly from the Chinese population. To date, in the Malaysian population, known for its ethnic diversity and multitudinous food choices, this is the first study aimed to determine the association between serum 25(OH)D and the risk of NPC, as well as its influence on survival among NPC cases.

## 2. Methodology

### 2.1. Study Subjects and Locations

A total of 300 histopathologically confirmed NPC cases and 300 matched cancer-free controls were recruited from two public hospitals. These hospitals were selected because they are large hospitals with heavy case workloads treating NPC cases and have been reported to have the highest admission of NPC cases of about 140 newly diagnosed cases per year. For each case recruited, one eligible control matched for age (±3 years), gender, and ethnicity was recruited consecutively from the general medical clinic and ward from the same hospital where the cases were treated.

### 2.2. Ethical Approval

Approval was obtained from the Ethics Committee for Research Involving Human Subjects, University Putra Malaysia (UPM/FPSIVPADS/T7-MJKElikaPer/Fo1) and the Medical Research and Ethics Committee of Ministry of Health (MREC; NMRR-11-1038-10007). Written informed consent was obtained from all participants prior to their participation. This study was performed in accordance with all the relevant guidelines and regulations as per the Institutional Ethical Committee and the Medical Research and Ethics Committee.

### 2.3. Test Principle of Total Serum 25(OH)D

Twenty milliliters of fasting venous blood was collected from each study subject. The blood was transferred into royal blue trace element serum tubes which contained spray-dried silica as a clotting aid. Upon transfer, the tubes were inverted 8 to 10 times to mix the clotting aid and the blood. Then, the specimens were allowed to sit at room temperature for 30 minutes before being centrifuged at 3000 rpm for 10 minutes. The sera were immediately poured into a trace element-free Eppendorf tube within six hours of collection and stored frozen (−80 °C) till the day of analysis.

Total serum 25(OH)D was measured using the LIAISON^®^ 25(OH)D TOTAL Assay, using commercially available kits from DiaSorin (Stillwater, MN, USA), which uses direct competitive chemiluminescent immunoassay (CLIA) technology. The analysis was conducted using a LIAISON^®^ Analyser in Gribbles Pathology Laboratory, Petaling Jaya, Selangor, Malaysia. The intra- and inter-assay CVs of the Liaison method were calculated by analyzing 10 duplicated serum samples over 3 days. The intra- and inter-assay % CV ranged from 3.3% to 8.04% and 6.7% to 11.7%, respectively.

To ensure appropriate test performance, the operating instructions of the LIAISON^®^ Analyzer were strictly adhered to. All important reagent preparations were handled pre-cautiously, especially during re-suspension of magnetic particles that were coated with antibody against 25(OH)D. To ensure complete suspension, before the seal was removed, the small wheel was rotated at the magnetic particle compartment until the color of the suspension was completely changed to brown, and foaming of reagents was avoided. Before the analysis, frozen sera were thawed and mixed well. Sera with lipemia, turbidity, particulate matter, or erythrocyte debris were cleared via filtration and centrifugation before testing. Approximately 75 μL of clear serum was transferred into a separate labeled glass tube and 225 μL of specimen diluent was added (1:3 dilution). This was to dilute samples with high 25(OH)D values into the assay range. Both samples and controls were vortexed gently before testing. Then, 250 μL of samples, calibrators, and controls were dispensed in respective reaction modules, followed by 25µL of suspended magnetic particles and assay buffer in every reaction module and incubated for 10 minutes. During the first incubation, 25(OH)D is dissociated from its binding protein and binds to the specific antibody in the solid phase. Then, 25 μL of tracer (isoluminol derivative) tagged to vitamin D was added and incubated for another 10 minutes. The unbound material was removed with a wash cycle subsequently; the starter reagents were added to initiate a flash chemiluminescent reaction. The light signal was measured with a photomultiplier as relative light units (RLUs) and it was inversely proportional to the concentration of serum 25(OH)D present in calibrators, controls, or samples. The LIAISON^®^ Analyzer calculates serum 25(OH)D concentrations in the sample automatically and expressed in nmol/L.

Since vitamin D has many physiological functions beyond bone, an optimal threshold for serum 25(OH)D sufficiency is still controversial. However, experts have suggested 25(OH)D concentrations of 30 ng/mL (75 nmol/L) and higher as an optimal threshold to ensure vitamin D adequacy to meet both bone and metabolic requirements [28]. The suggestion is substantiated by various studies, indicating that serum levels of 25(OH)D at 30 ng/ml and above are linked to enhanced cardiometabolic health and a reduced risk of specific chronic diseases [29,30,31,32,33,34,35]. In this study, a serum 25(OH)D concentration lower than 30 ng/mL was considered an inadequate serum 25(OH)D status.

### 2.4. Interviewer-Administered Questionnaire

Face-to-face interviews were conducted for study subjects (cases and controls) after obtaining written consent. The pre-tested questionnaire included questions on (i) socio-demographic data such as age, sex, and ethnicity (Malay, Chinese, or Indian); (ii) smoking habit; (iii) alcohol consumption; (iv) intensity of physical activity using the Global Physical Activity Questionnaire (GPAQ) per week); (v) weekly consumption patterns of foods high in vitamin D such as beef liver, pork, fish, egg, soy products, and dairy products using a semi-quantitative food frequency questionnaire (SFFQ); (vi) body mass index (BMI; weight (kg)/height^2^ (m^2^)); and (vii) salted fish consumption.

### 2.5. Data Analysis

Descriptive statistics, including frequencies, percentages (%), means with standard deviations (µ ± SD), and median with interquartile range (IQR), were applied for data description. Categorical variables were presented as absolute numbers and percentages. Normally distributed variables were reported as mean ± SD, while non-parametric methods were employed for non-normally distributed values. To assess differences between the case and control groups for continuous variables, either the Mann–Whitney U test or independent *t*-test and ANOVA were used. The association between categorical variables was determined using the Chi-square (χ^2^) distribution. The association between serum 25(OH)D and NPC risk was determined using multiple conditional logistic regression analysis with IBM SPSS. The association was reported in odds ratios (ORs) with 95% confidence intervals (95% CI). To adjust for possible confounding, the following NPC risk factors were included as covariates in the multiple variable models: body mass index, intensity of physical activity, consumption of foods highest in vitamin D, smoking status, alcohol consumption, salted fish consumption, and family history of NPC. Polynomial regression analysis was conducted to assess the association between inadequate serum 25(OH)D with the addition of each risk factor and NPC. The trend line was added in Microsoft Excel 2021 (v16.0) to generate the equation for the line of best fit and coefficient of determination (R^2^). Cox proportional hazards regression analysis was conducted to investigate the effect of serum 25(OH)D on survival of NPC patients. 

## 3. Results

### 3.1. Characteristics of Study Subjects

The mean age of cases and controls were 54.06 ± 10.94 and 54.24 ± 11.11 years old, respectively. Table 1 shows the relative proportions for the age group of 50 to 59 years old were 35%, followed by 25.7% for those aged 40 to 49 years old, 24.3% for 60 to 69 years old, and 9.3% for those aged below 40 years old. The proportion of males diagnosed with NPC was higher than the females (77.0% vs. 23.0%, respectively). Chinese made up 71.0% of cases. Slightly more than half (52.7%) of the cases had normal BMI, while 20% of them were underweight and 20% were overweight. Although the majority of the controls had normal BMI (39.7%), the proportion of the controls who were overweight or obese was higher than in cases (33.3% and 22.7%, respectively). The median BMI of cases was significantly lower than the controls (21.62 kg/m^2^ vs. 25.93 kg/m^2^). The median physical activity frequency was significantly higher among cases compared to controls (85.3 h/week vs. 69.7 h/week). A significantly higher proportion of cases (51.7%) had ever smoked, compared with controls. The proportion of cases that never consumed alcohol was lower than the controls (54.3% vs. 62.3%, respectively), but this was not significant. There was no association between consumption of high vitamin D content food such as beef, pork, fish, and dairy products and NPC; however, consumption of egg and soy products once a week or more was significantly higher among cases compared to controls (8.7% vs. 2.7%, χ^2^ = 10.1, *p* = 0.001 and 16.7% vs. 7.7%, χ^2^ = 11.37, *p =* 0.001, respectively). A significantly higher proportion of cases ever consumed salted fish (76.0% vs. 57.7%, χ^2^ = 22.75, *p* < 0.001). The median amount of salted fish intake was significantly higher among cases compared to controls (1.88 g/day vs. 0.66 g/day). The proportion of cases with a family history of NPC was significantly higher among cases than controls (12.3% vs. 2.0%, *p* < 0.001) (Table 1).

### 3.2. Distribution of 25(OH)D Serum Concentration across Clinical Characteristics of Nasopharyngeal Carcinoma Cases

Table 2 shows the 25(OH)D serum concentration for cases across clinical characteristics of NPC. The findings show that there is no difference in 25(OH)D serum concentration across the clinical characteristics of NPC, except for radiotherapy treatment. The 25(OH)D serum concentration was higher among those who underwent radiotherapy (25.64 ± 7.63 ng/mL vs. 21.21 ± 7.09; *p* = 0.034).

### 3.3. Association between the Serum Concentration of 25(OH)D and Nasopharyngeal Carcinoma Adjusting for Confounding Factors

There was a significant inverse association between the serum concentration of 25(OH)D and the odds of developing NPC after adjusting for confounding (AOR = 0.73, 95% CI = 0.57–0.94, *p* = 0.016). Being underweight, salted fish consumption, and having a family history of NPC independently increased the odds of NPC. Having a family history of NPC was significantly directly associated with NPC more than sixfold (AOR = 6.59, 95% CI = 2.63, 16.56, *p* < 0.001). NPC cases had a fivefold likelihood of being underweight compared to controls (AOR = 5.82, 95% CI = 2.79, 12.14, *p* < 0.001). The consumption of salted fish independently doubled the odds of NPC (AOR = 2.40, 95% CI = 1.58, 3.65, *p* < 0.001). Lifestyle factors such as smoking and alcohol consumption showed no independent association with NPC (Table 3).

### 3.4. Dose–Response Analysis between Inadequate Serum Concentration of 25(OH)D with Accumulation of Risk Factors and Nasopharyngeal Carcinoma Using Polynomial Analysis

**Figure 1:**
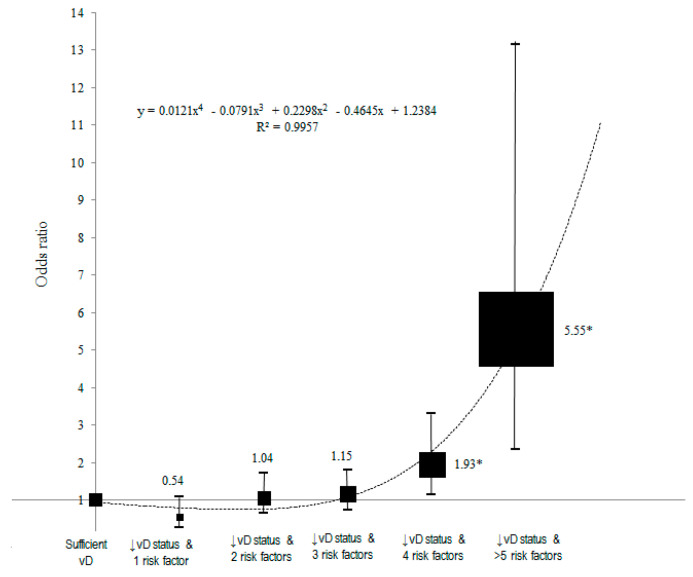
ORs for NPC versus cumulative effect of inadequate serum 25-hydroxyvitamin D status with number of risk factors using polynomial regression for dose–response analysis. Note: (*) statistically significant; (↓vD) inadequate serum 25(OH)D.

Figure 1 shows the risk of NPC using polynomial regression analysis, where the resulting function OR(x) = 0.0121x^4^ − 0.0791x^3^ + 0.2298x^2^ − 0.4645x + 1.2384 shows a non-linear association, setting those with adequate serum 25(OH)D (>30 ng/mL) from the categorical analysis as reference. The black squares and lines indicate the odd ratios and 95% confidence intervals of the ordinal variables. The size of the black square indicates the effect size of the association at every step. A good fit was shown between the curve and the odds ratio estimates of the categorical analysis (R^2^ = 0.9957). Inadequate serum 25(OH)D status with the accumulation of each risk factor showed a dose–response association with odds of getting NPC. There was a significant association between inadequate serum 25(OH)D status with accumulation of four risk factors and increased odds ratio of getting NPC. An increased NPC odds ratio was observed after sequential accumulation of additional risk factors with the presence of inadequate serum 25(OH)D status (OR = 0.54, 95% CI = 0.27, 4.77, *p* = 0.322, OR = 1.04, 95% CI = 0.64, 1.72, *p* = 0.267, OR = 1.15, 95% CI = 0.73, 1.80, *p* = 0.067, OR = 1.93, 95% CI = 1.13, 3.31, *p* = 0.022, and OR = 5.55, 95% CI = 1.67, 10.3, *p* < 0.001, respectively).

### 3.5. Five-Year Survival Time of Nasophrayngeal Carcinoma Cases across the Levels of Serum 25(OH)D Concentration

**Figure 2:**
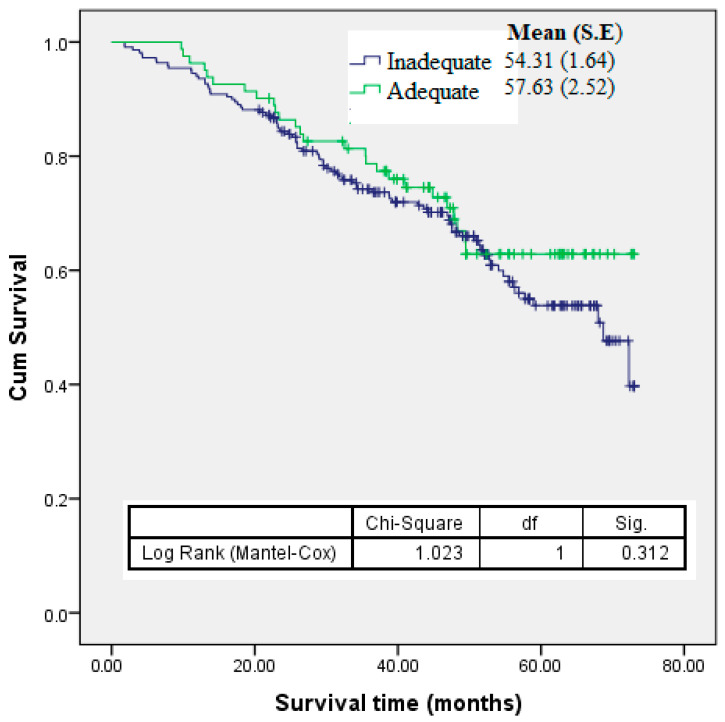
Kaplan-Meier 5-year cumulative survival curves of NPC cases for different levels of serum 25-hydroxy vitamin D.

Figure 2 shows the Kaplan–Meier estimates for the 5-year survival time of NPC cases across the levels of serum 25(OH)D. The mean 5-year survival rates were 54.5% for cases with inadequate serum 25(OH)D and 57.6% for cases with adequate serum 25(OH)D. However, no significant association was found between levels of serum 25(OH)D and survival of NPC (AHR = 0.94, 95% CI = 0.77, 1.15, *p* = 0.550).

## 4. Discussion

In line with strategic research directions of the World Cancer Research Fund International [36], this hospital-based case–control study has addressed an important question on the role of vitamin D deficiency, a global nutritional problem, in NPC. Of note, this is the first study evaluating the association between serum 25(OH)D and NPC specifically in the Malaysian population. In this study, the mean serum 25(OH)D levels were inadequate in both cases and controls, being slightly lower among cases compared to controls. There was a serious deficiency in mean serum 25(OH)D in patients with head and neck cancer (16.8 ng/mL)—20% of the patients had inadequate serum 25(OH)D [37] while 45% of the patients had serum 25(OH)D deficiency [37]. In this study, there is no significant difference in serum 25(OH)D across clinical characteristics of NPC, except between those who received radiotherapy and those who did not. Interestingly, serum 25(OH)D was significantly higher among those who received radiotherapy. The association observed may not imply causation, but, potentially, individuals undergoing radiotherapy may be more likely to take vitamin D supplements or receive dietary recommendations to support their overall health during treatment. Radiotherapy may have direct or indirect effects on vitamin D metabolism or absorption [38], which warrant further investigation.

This study found that higher concentrations of serum 25(OH)D were significantly associated with a lower odds ratio of NPC, where an increase of 1 ng/mL of serum 25(OH)D reduced the odds ratio of NPC by 27%. This finding shows that inadequate serum 25(OH)D is independently associated with NPC. A nested case–control study by the European Prospective Investigation into Cancer and Nutrition (EPIC) study also showed an inverse association; the risk of head and neck cancer was reduced by 30% with an increase of 1 ng/mL of serum 25(OH)D. A significant inverse association was observed, especially with the risk of larynx or hypopharynx cancer (OR = 0.55, 95 CI% = 0.39–0.78) and oral cancer (OR = 0.60, 95 CI% = 0.42–0.87) [39]. In in-vivo and in-vitro cancer models, 25(OH)D reduces angiogenesis, tumor invasion and metastasis in head and neck squamous cells [40], and oral [41] and thyroid [42] cancer. Angiogenesis and tumor invasion and metastasis are important mechanisms associated with the survival of cancer cells [34]. However, the current study showed no association between serum 25(OH)D level and survival of NPC.

The protective effect of vitamin D on cancer development may be explained by its anti-inflammatory, anti-proliferative, anti-apoptosis, and angiogenesis suppressive mechanisms [43,44]. In cancer-related chronic inflammation, serum 25(OH)D is converted excessively intracellularly into 1,25-hydroxyvitamin D to activate the Vitamin Dreceptor (VDR) gene. In the cancer cell, the VDR gene is up-regulated in response to tumor progression and then down-regulated as the tumor cell de-differentiates. As the tumor progresses, the rate of VDR translocation to the nucleus is reduced and the response of the tumor to the action of the VDR gene is reduced [45,46]. It has been reported that inadequate serum 25(OH)D is a biomarker of the presence of chronic inflammation in NPC [12].

This study found that inadequate serum 25(OH)D with increasing accumulation of risk factors showed a dose–response effect of increasing odds of getting NPC, as shown in Figure 2. This generation of a risk model between well-established risk factors of NPC may provide an opportunity to improve health services. These services could be delivered to those with clusters of risk factors rather than having to separate care into different services targeted at individual risk factors to reduce the incidence of chronic diseases. In this study, inadequate serum 25(OH)D status with an increased number of risk factors showed a positive dose–response with the odds ratio of NPC, especially after the accumulation of a second risk factor. This is the first study illustrating the risk contribution for NPC as a cluster of risk factors with inadequate serum 25(OH)D status. The current results may form a basis for future studies to determine whether there are common pathophysiologic mechanisms underlying this cluster of risk factors, which will be useful as biomarkers for NPC risk assessment, screening, prognosis, and treatment. If these mechanisms can be clarified, they may present opportunities for the development of therapeutic options treating multiple traits simultaneously.

One of the limitations of this study is that serum 25(OH)D was measured at the time of diagnosis of NPC, so the association that was detected could be due to reverse causality. The nutritional condition due to the cancer and its treatments (for example, cachexia, nausea, vomiting, or anorexia) may alter the nutrient intake and absorption, which may have affected the 25(OH)D serum concentration. Recall bias in food intake using SFFQ is another limitation usually resulting in over-reporting of the quantity of food consumption. To minimize this bias, all the data were collected by trained interviewers using standardized interviewer-administered questionnaires. We also included memory aids such as food albums and household measurement tools. The third limitation is differential reporting bias because of the tendency of respondents to give a good impression of their diet and lifestyle practices by not reporting on unhealthy food consumption behaviors.

## 5. Conclusions

The study identifies an inverse association between serum 25(OH)D concentration and the odds ratio of NPC. Although the literature on 25(OH)D concentrations in head and neck cancers is limited, the consistency of findings in various cancer types such as colorectal cancer and prostate cancer further supports the findings of this study. It is noteworthy that the study did not establish a significant association between serum 25(OH)D and NPC survival. Future research should include prospective cohort studies and randomized controlled trials (RCTs) on the Malaysian population to validate and confirm these findings and further elucidate the role of vitamin D in NPC outcomes.

## Figures and Tables

**Table 1:** Characteristics of study subjects.

Variables		Case		Control		χ^2^	*p*-Value
		*n* (%)	Mean ± SD/ Median (IQR) ^b^	*n* (%)	Mean ± SD/ Median (IQR) ^b^		
**Age of respondents (years)**			54.06 ± 10.94		54.24 ± 11.11		
<40		28 (9.3)		27 (9.0)		1.97	0.741
40–49		77 (25.7)		71 (23.7)			
50–59		105 (35.0)		96 (32.0)			
60–69		73 (24.3)		86 (28.7)			
≥70		17 (5.7)		20 (6.7)			
**Gender**							
Male		231 (77.0)		231 (77.0)		-	-
Female		69 (23.0)		69 (23.0)			
**Ethnicity**							
Malay		85 (28.3)		85 (28.3)		-	-
Chinese		213 (71.0)		213 (71.0)			
Indian		2 (0.7)		2 (0.7)			
**Body mass index (kg/m^2^)**			21.62 (6.06) ^b^		25.93 (6.56) ^b^	−9.55 ^a^	<0.001 *
Underweight	<18.5	60 (20.0)		13 (4.3)		69.26	<0.001 *
Normal	18.5–24.9	158 (52.7)		119 (39.7)			
Overweight	25–29.9	60 (20.0)		100 (33.3)			
Obese	≥30	22 (7.3)		68 (22.7)			
**Physical activity intensity** **(MET; hour/week)**			85.3 (184.50) ^b^		69.7 (128.50) ^b^	2.03 ^a^	0.043 *
**Smoking status**							
Ever smoke		155 (51.7)		126 (42.0)		5.63	0.018 *
Never smoke		145 (48.3)		174 (58.0)			
**Alcohol consumption**							
Ever consumed		137 (45.7)		114 (38.0)			
Never consumed		163 (54.3)		186 (62.0)			
**Consumption of food high in vitamin D**							
** Beef_Liver**							
<once a week		65 (21.7)		58 (19.3)		0.50	0.479
≥once a week		235 (78.3)		242 (80.7)			
** Pork**							
<once a week		193 (64.3)		202 (67.3)		0.60	0.439
≥once a week		107 (35.7)		98 (32.7)			
** Fish**							
<once a week		291 (97.0)		296 (98.7)		1.97	0.161
≥once a week		9 (3.0)		4 (1.3)			
** Egg**							
<once a week		274 (91.3)		292 (97.3)		10.10	0.001 *
≥once a week		26 (8.7)		8 (2.7)			
** Soy products**							
<once a week		250 (83.3)		277 (92.3)		11.37	0.001 *
≥once a week		50 (16.7)		23 (7.7)			
** Dairy products**							
<once a week		248 (82.7)		254 (84.7)		0.44	0.508
≥once a week		52 (17.3)		46 (15.3)			
** Salted fish consumption**							
Ever		228 (76.0)		173 (57.7)		22.75	<0.001 *
Never		72 (24.0)		127 (42.3)			
** Consumption of salted fish per day (g)**			1.88 (4.41) ^b^		0.66 (1.88) ^b^	−5.45 ^a^	<0.001 *
** Family history of NPC**							
Yes		37 (12.3)		6 (2.0)		24.07	<0.001 *
No		263 (87.7)		294 (98.0)			

Note: IQR = interquartile range; METs = metabolic equivalence; (^a^) non-parametric test using Mann–Whitney U; (^b^) median (IQR); (*) statistically significant.

**Table 2:** Serum 25-hydroxyvitamin-D across the clinical characteristics of nasopharyngeal carcinoma.

Clinical Characteristics	Mean ± SD	F/t	*p*-Value
**Cancer indicators ^++^**			
Neck swelling	26.19 ± 7.73	1.751 ^e^	0.081
Nasal symptoms	25.57 ± 7.49	−0.393 ^e^	0.695
Aural symptoms	25.35 ± 7.72	0.086 ^e^	0.931
Facial numbness	24.85 ± 7.35	−0.329 ^e^	0.743
Other symptoms	25.17 ± 7.41	−0.211 ^e^	0.833
**Histopathological grading ^a^**			
WHO type I = keratinising squamous-cell carcinoma	25.92 ± 6.94	0.209 ^f^	0.648
WHO type II = non-keratinising epidermoid carcinoma	25.44 ± 7.97		
WHO type III = undifferentiated carcinoma	25.56 ± 7.71		
**AJCC staging for NPC**			
**T= Tumor ^b^**			
0: no evidence of primary tumor	39.26 ± 1.23	1.063 ^f^	0.381
1: tumor confined to nasopharynx	24.93 ± 7.93		
2a: tumor extends to soft tissue of oropharynx and/or Nasal cavity without parapharyngeal extension	25.12 ± 7.66		
2b: tumor extends to soft tissue of oropharynx and/or Nasal cavity with parapharyngeal extension	26.61 ± 8.08		
3: tumor invading bony structures and/or paranasal sinuses	25.60 ± 6.74		
4: tumor with intracranial extension and/or involvement of cranial nerve			
**N = Regional nodes ^b^**			
0: none	25.30 ± 7.72	0.191 ^f^	0.943
1: unilateral nodal metastasis < 6 cm in greatest diameter	25.36 ± 7.83		
2: bilateral nodal metastasis < 6 cm in greatest diameter	25.58 ± 8.11		
3a: cervical lymph nodes > 6 cm	24.31 ± 5.91		
3b: any cervical nodes in superclavicular fossa	25.77 ± 5.91		
**M = Metastasis ^c^**			
0: no	25.22 ± 7.60	1.219 ^g^	0.224
1: yes	28.36 ± 7.86		
**Staging ^b^**			
1	24.58 ± 9.00	0.468 ^f^	0.800
2	24.75 ± 8.23		
3	25.91 ± 7.83		
4A	25.13 ± 7.38		
4B	24.32 ± 6.05		
4C	26.93 ± 8.68		
**Staging investigations procedures ^¥,d^**			
Endoscopy	25.11 ± 7.53	−1.099 ^e^	0.272
CT scan	25.56 ± 7.56	1.582 ^e^	0.295
MRI scan	26.84 ± 7.17	1.569 ^e^	0.118
Bone scan	26.58 ± 7.06	1.387 ^e^	0.166
Chest radiography	24.60 ± 6.56	−0.611 ^e^	0.541
USS liver	25.12 ± 7.72	−0.209 ^e^	0.834
PET scans	24.99 ± 6.40	−0.349 ^e^	0.295
**Treatment**			
**Radiotherapy**			
Yes	25.64 ± 7.63	2.125 ^g^	0.034 *
No	21.21 ± 7.09		
**Type of radiotherapy (n = 281)**			
External beam radiotherapy (EBRT)	25.69 ± 7.56	1.019 ^f^	0.398
Palliative radiotherapy	20.03 ± 12.47		
Autologous SCT	21.92 ± 6.74		
Deep X-ray therapy	37.37 ± 2.13		
Unknown	26.31 ± 7.40		
**Chemotherapy**			
Yes	25.33 ± 7.68	0.130 ^g^	0.897
No	25.18 ± 7.71		
**Type of chemotherapy (N = 253)**			
Induction	26.06 ± 7.07	0.475 ^f^	0.752
Adjuvant	24.49 ± 4.36		
Concurrent	24.91 ± 8.45		
Palliative	25.35 ± 4.20		
Unknown	23.12 ± 5.35		
**Surgery**			
Yes	27.75 ± 7.98	1.312 ^g^	0.191
No	25.17 ± 7.65		
**Type of surgery (N = 16)**			
Debulking nasopharynx	34.11 ± 4.39	0.509 ^f^	0.731
Radical neck dissection	28.73 ± 7.50		
Nasopharyngectomy	27.67 ± 10.70		
Unknown	28.47 ± 11.19		
**Response to treatment**			
Complete remission	25.04 ± 7.70	−1.209 ^g^	0.228
Diagnosed with residual disease	26.36 ± 7.55		
**Survival status**			
Alive	25.50 ± 7.77	0.581 ^g^	0.562
Not alive	24.97 ± 7.52		

Note: (^a^) = 15 (5.0%) missing; (^b^) = 13 (4.3%) missing; (^c^) = 12 (4.0%) missing; (^d^) = 11 (3.7%) missing; (^e^) = multiple response variables, with the mean compared between the presence and absence of each item using independent *t*-test; (^f^) F= one-way ANOVA; (^g^) t = independent *t*-test; (++) = the mean serum 25(OH)D concentration in cases where each indicator is present; (^¥^) = the mean serum 25(OH)D concentration in cases that underwent each staging investigations procedure; (*) statistically significant.

**Table 3:** Univariable analysis and multiple conditional logistic regression for risk factors of nasopharyngeal carcinoma.

Factors	Case *n* (%)	Control *n* (%)	COR ^+^ (95% CI)	*p*-Value	B	SE	AOR ^++^ (95% CI) ^a^	*p*-Value
**Serum 25(OH)D** **(Mean ± SD)**	25.3 ± 7.7	27.0 ± 9.2	0.78 (0.66, 0.95)	0.011 *	−0.029	0.013	0.73 (0.57, 0.94)	0.016 *
**Body mass index**								
<18 kg/m^2^	60 (20.0)	13 (4.3)	6.22 (3.08, 12.58)	<0.001 *	1.74	0.38	5.82 (2.79, 12.14)	<0.001 *
≥18 kg/m^2^	240 (80.0)	287 (95.7)	1 ^b^				1 ^b^	
**Salted fish consumption**								
Ever	228 (76.0)	173 (57.7)	2.34 (1.62, 3.38)	<0.001 *	0.88	0.22	2.40 (1.58, 3.65)	<0.001 *
Never	72 (24.0)	127 (42.3)	1 ^b^				1 ^b^	
**Family history of NPC**								
Yes	37 (12.3)	6 (2.0)	6.17 (2.60, 14.61)	<0.001 *	1.92	0.47	6.59 (2.63, 16.56)	<0.001 *
No	263 (87.7)	294 (98.0)	1 ^b^				1 ^b^	
**Smoking**								
Ever smoked	155 (51.7)	126 (42.0)	1.69 (1.15, 2.48)	0.007 *	0.31	0.23	1.35 (0.86, 2.11)	0.174
Never smoked	145 (48.3)	174 (58.0)	1 ^b^				1 ^b^	
**Alcohol consumption**								
Ever consumed	137 (45.7)	114 (38.0)	1.55 (1.05, 2.28)	0.027 *	0.19	0.24	1.22 (0.77, 1.93)	0.413
Never consumed	163 (54.3)	186 (62.0)	1 ^b^				1 ^b^	
**Physical activity**								
Low intensity (MET < 10 hours/week)	256 (85.3)	251 (83.7)	1.13 (0.73, 1.75)	0.579	na	na	na	na
High intensity (MET ≥ 10 hours/week)	44 (14.7)	49 (16.3)	1 ^b^					
**Consumption of food high in Vitamin D**								
<once a week	128 (42.7)	128 (42.7)	1.00 (0.72, 1.40)	1.000	na	na	na	na
≥once a week	172 (57.3)	172 (57.3)	1 ^b^					

Note: (^a^) age, gender, and ethnicity have been controlled for in the design stage by matching; (^b^) reference category; NPC = nasopharyngeal carcinoma; (*) statistically significant; ^+^ COR = crude odds ratio; ^++^ AOR = adjusted odds ratio.

## Data Availability

All data for this study are available upon reasonable request from the corresponding author.
